# Supplementary material for: Cyclodextrin-Based Nanoparticles for Delivery of Antisense Oligonucleotides Targeting Huntingtin
Source: Pharmaceutics. 2023 Feb 3;15(2):520. doi: 10.3390/pharmaceutics15020520 (PMC9965918; doi:10.3390/pharmaceutics15020520)
Supplement: Supplementary file 1 [file pharmaceutics-15-00520-s001.zip › pharmaceutics-2145140-supplementary.pdf]

pharmaceutics

MDPI

Article

Cyclodextrin-based nanoparticles for delivery of antisense oligonucleotides targeting Huntingtin

Monique C. P. Mendonça [1], Yao Sun [1], Michael F. Cronin [1], Andrew J. Lindsay [2], John F. Cryan [3] and Caitriona M. O'Driscoll [1,*]

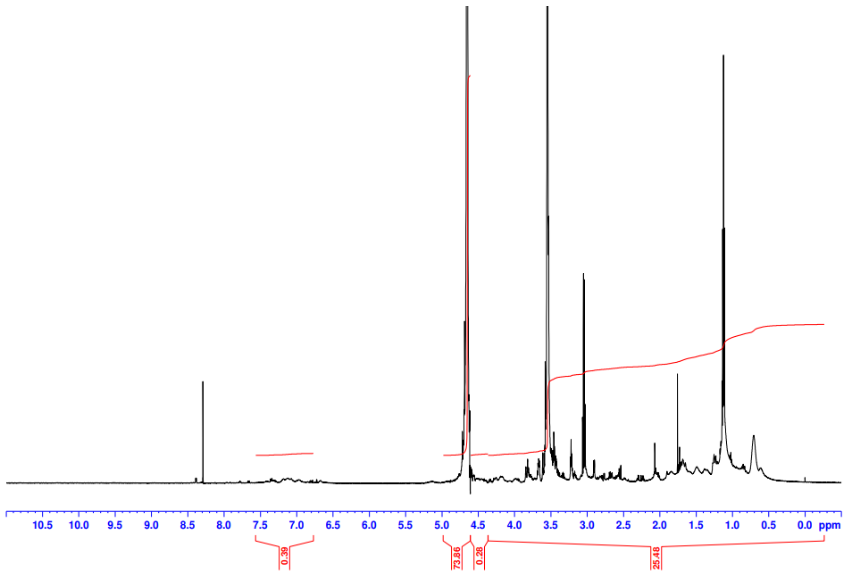

Figure S1. ^{1}H NMR spectra of DSPE-PEG-Maleimide-RVG product in D_2O. The loss of the characteristic maleimide peak at 6.68 ppm confirmed the successful conjugation of RVG to maleimide-CD.

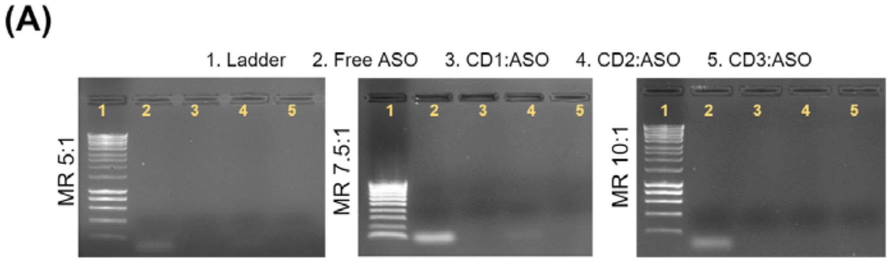

(B)

		WR 5:1	WR 7.5:1	WR 10:1
**CD1:ASO**	Z-average (nm)	106 ± 3.14	150 ± 8.60	175 ± 7.14
	PDI	0.29 ± 0.03	0.32 ± 0.01	0.32 ± 0.03
	Zeta potential (mV)	4 ± 0.38	12 ± 0.833	11 ± 0.24
**CD2:ASO**	Z-average (nm)	1520 ± 121.3	807 ± 36.95	174 ± 2.73
	PDI	0.37± 0.09	0.17 ± 0.04	0.36 ± 0.03
	Zeta potential (mV)	-39 ± 0.90	0.6 ± 60	5 ± 0.30
**CD3:ASO**	Z-average (nm)	105 ± 2.85	131 ± 5.45	148 ± 3.33
	PDI	0.21 ± 0.01	0.21 ± 0.01	0.26 ± 0.03
	Zeta potential (mV)	9 ± 0.38	13 ± 1.08	15 ± 0.20

Figure S2. (A) Representative agarose gel electrophoresis illustrating ASO binding to cyclodextrins (CDs); (B) Physicochemical properties of CDs: ASO nanocomplexes at different weight ratios (WR).

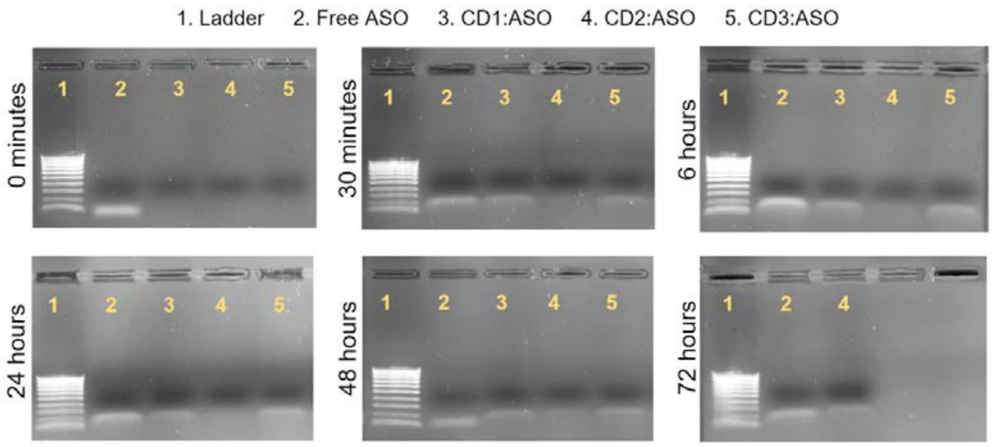

Figure S3. Representative agarose gel electrophoresis illustrating heparin-induced ASO release from CDs: ASO complexes.

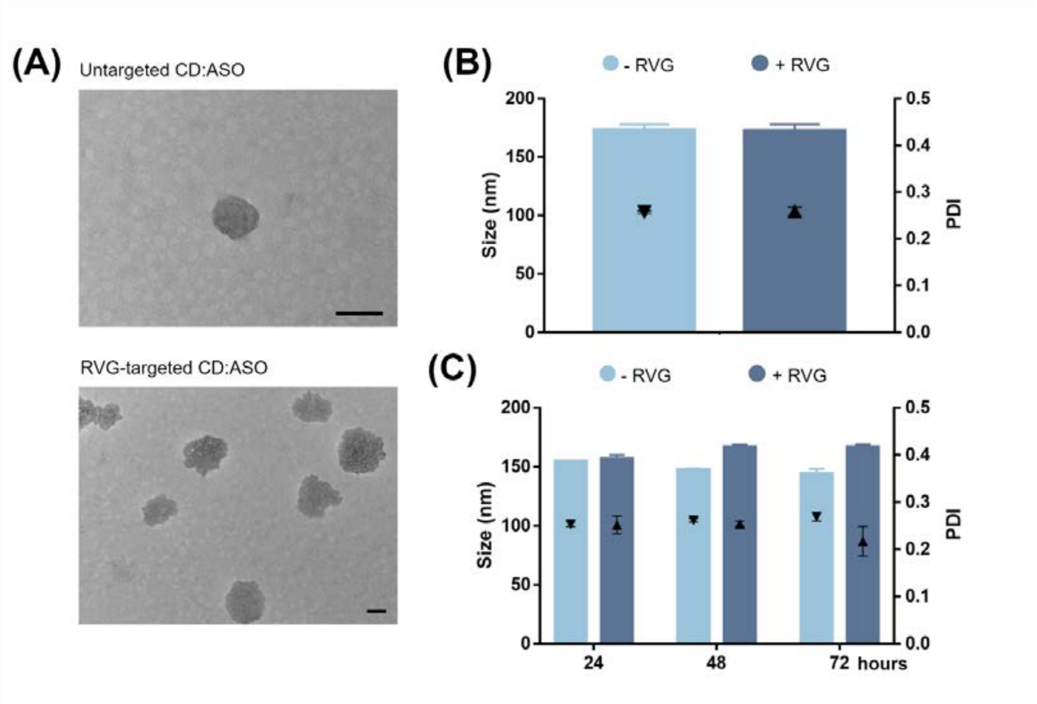

Figure S4. **Physicochemical characterisation of RVG-targeted cyclodextrins (CD).ASO complexes.** (A) Representative transmission electron microscopy (TEM) of RVG-targeted and untargeted CD3:ASO nanocomplexes. (B) Particle size and *Polydispersity index* (PDI) in hCMEC/D3 culture media. (C) Time-dependent particle size and PDI in hCMEC/D3 culture media at 37°C. Mean ± S.E.M of 6 technical replicates. All at mass ratio 10:1, 750 nM ASO. Scale bars = 100 nm.

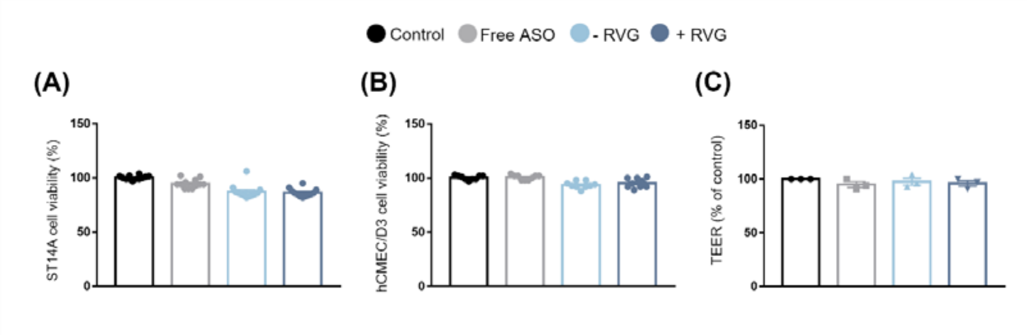

Figure S5. Effects of RVG-targeted cyclodextrins (CD)s.ASO complexes on cell viability and TEER. (A) The viability of ST14A cells (1.5 x 10^6) and hCMEC/D3 cells (3.0 x 10^4) were measured 72 h after treatment with CDs loaded with ASO, weight ratio 10:1, 100 nM per well. Living cells were determined using a CellTiter-FLuor assay and expressed as a % of the control. (B) Transendothelial resistance (TEER) was measured on day 11 (3 days of treatment) using EVOL volt-ohm meter. TEER values are shown as % of control. Mean ± S.E.M of 3 technical replicates performed in triplicate.

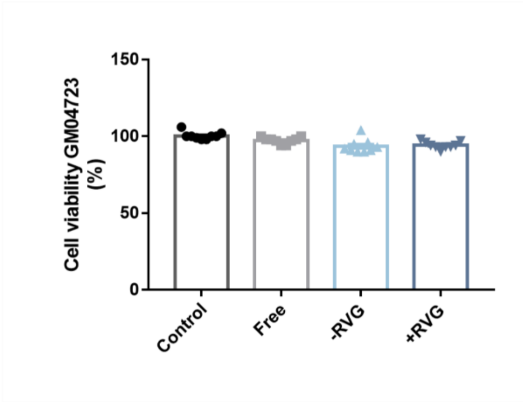

Figure S6. Viability of HD patient-derived fibroblast cell line GM04723 (CAG15/67) measured 72 h after treatment with CDs loaded with ASO, weight ratio 10:1, 100 nM per well. Living cells were determined using a CellTiter-FLuor assay and expressed as a % of the control. Mean ± S.E.M of 3 technical replicates performed in triplicate.

Table S1. Results of two-way analysis of variance of the effects of RVG-targeted and untargeted cyclodextrins: ASO nanocomplexes on total HTT expression.

	Effect	p	F
**Co-culture**	Targeting	0.0017	7.028
	Mutation	< 0.0001	28.16
	Interaction	0.0013	7.03
**Monoculture**	Targeting	< 0.0001	38.77
	Mutation	0.0083	8.79
	Interaction	ns	1.54
